# Stromal Protein Ecm1 Regulates Ureteric Bud Patterning and Branching

**DOI:** 10.1371/journal.pone.0084155

**Published:** 2013-12-31

**Authors:** Suneeta S. Paroly, Fengwei Wang, Lee Spraggon, Joseph Merregaert, Ekatherina Batourina, Benjamin Tycko, Kai M. Schmidt-Ott, Sean Grimmond, Melissa Little, Cathy Mendelsohn

**Affiliations:** 1 Department of Urology, Irving Cancer Research Center, Columbia University, New York, New York, United States of America; 2 Institute for Cancer Genetics & Taub Institute for Research on Alzheimer’s Disease and the Aging Brain, Columbia University, New York, New York, United States of America; 3 Laboratory of Molecular Biotechnology, Department of Biochemistry, University of Antwerp, Wilrijk, Belgium; 4 Max-Delbrueck Center for Molecular Medicine Robert-Roessle-Str. Berlin, Germany; 5 Institute for Molecular Bioscience, The University of Queensland St Lucia QLD, Australia; Tulane University School of Medicine, United States of America

## Abstract

The interactions between the nephrogenic mesenchyme and the ureteric bud during kidney development are well documented. While recent studies have shed some light on the importance of the stroma during renal development, many of the signals generated in the stroma, the genetic pathways and interaction networks involving the stroma are yet to be identified. Our previous studies demonstrate that retinoids are crucial for branching of the ureteric bud and for patterning of the cortical stroma. In the present study we demonstrate that autocrine retinoic acid (RA) signaling in stromal cells is critical for their survival and patterning, and show that *Extracellular matrix 1*, *Ecm1*, a gene that in humans causes irritable bowel syndrome and lipoid proteinosis, is a novel RA-regulated target in the developing kidney, which is secreted from the cortical stromal cells surrounding the cap mesenchyme and ureteric bud. Our studies suggest that Ecm1 is required in the ureteric bud for regulating the distribution of *Ret* which is normally restricted to the tips, as inhibition of Ecm1 results in an expanded domain of *Ret* expression and reduced numbers of branches. We propose a model in which retinoid signaling in the stroma activates expression of *Ecm1*, which in turn down-regulates *Ret* expression in the ureteric bud cleft, where bifurcation normally occurs and normal branching progresses.

## Introduction

The adult metanephric kidney is a complex organ composed of nephrons, collecting ducts and blood vessels. The development of the metanephric kidney involves reciprocal molecular interactions between ureteric bud cells, the nephrogenic mesenchymal cells and the stromal mesenchymal cells [1]–[4]. Branching of the ureteric bud and induction of nephrons depend on bidirectional signaling between the invading ureteric bud and the surrounding metanephric mesenchyme. In addition to ureteric bud-nephron progenitor signaling, a number of studies indicate that signals from cortical stromal cells are also required for maintaining nephron differentiation and branching morphogenesis, including *Foxd1*, a winged helix transcription factor, *Pod1* and *Hox* genes [3], [5]–[9].

The GDNF/Ret signaling cascade is central to ureteric bud outgrowth and normal branching, where *Ret* signaling in the ureteric bud tip induced by Gdnf secreted from nephron progenitors, controls cell rearrangements and/or migration that are critical for branching morphogenesis reviewed in: [10]–[12]. Studies from our lab and others indicate that retinoic acid (RA), a potent transcriptional activator which is secreted from cortical stroma, is required for maintaining expression of *Ret* in ureteric bud tips [13], [14]. We further showed that RA-regulates *Ret*-in a cell autonomous manner, by activating RA-receptor signaling in the ureteric bud [15]. Recent studies suggest that RA-receptors can directly regulate *Ret* transcription in combination with Estrogen receptors via enhancers located in *Ret* regulatory sequences [16].

To evaluate whether RA-signaling has additional roles in renal development, we performed gene expression profiling in stromal cells isolated from embryonic kidneys cultured with or without RA. Our studies identified a number of known RA-targets and several novel RA-regulated genes including *Extracellular matrix protein 1* (hereafter *Ecm1*), a gene that when mutated in humans causes lipoid proteinosis and ulcerative colitis [17], [18]. We show that inhibition of *Ecm1* activity results in ectopic expression of *Ret* in the clefts of the ureteric bud branches and fewer, abnormal branches of the ureteric bud. Therefore our study identifies a novel function of the retinoic acid induced signaling pathway in cortical stroma that is important for *Ret* expression and branching morphogenesis.

## Results

**Figure 1:**
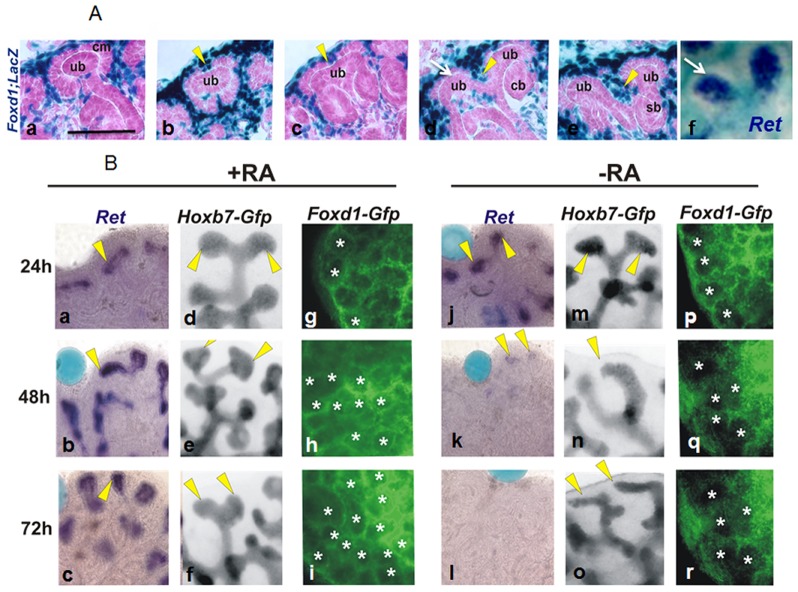
A) Stromal cell distribution depends on *Ret* and branching. Histological sections of Foxd1-LacZ kidneys during embryonic development showing the distribution of stromal cells (blue) in the cortical region of the kidney (a–f). (a) Ampulla stage of ureteric bud (shown in pink, ureteric bud, ub) surrounded by nephron progenitor cells (pink, condensing mesenchyme, cm) and Foxd1- lacZ stromal cells (blue). (b) Growing ampulla surrounded by nephron progenitors at the tips and cleft (yellow arrow head) at the center occupied by stromal cells. (d–e) Stromal cells occupy the cleft made by the bifurcating ureteric bud tips. (f) *Ret* expression in the ureteric bud tips (white arrows). B) In vitro cultures of Hoxb7-GFP and Foxd1-GFP embryonic kidneys grown either in the presence of RA (culture beads with 10 µg/mL of RA) (a–i) or in the absence of RA (culture beads with basal media) (j–r) for 24, 48 and 72 hours. Hoxb7-GFP kidney cultures were probed for *Ret* expression after culture at 24, 48 and 72 hours (a–c and j–l). Hoxb7-GFP kidneys showing ureteric bud branching (d–f and m–o). Foxd1-GFP kidneys showing stromal cell distribution (g–i and p–r), asterisks show the ureteric buds with stromal cells around them.

In mutants lacking *Rars* or *Raldh2*, stromal cells accumulate at the periphery of the embryonic kidney and do not assume their normal position surrounding induced nephons and ureteric bud (ub) tips [15], [19]. Studies in the lung suggest that the cleft formation during branching is controlled by extracellular matrix (ECM)-components secreted by surrounding mesenchymal cells [20]. To look at the relative positions of stroma and ureteric bud during branching, we analyzed embryonic kidneys from *Foxd1-lacZ* embryos in which lacZ is selectively expressed in cortical stroma [5]. At the ampulla stage, ub tips in *Foxd1-lacZ* embryos are surrounded by cap mesenchyme, and cortical stromal cells are excluded from direct contact with the tip (Fig. 1 A a). As the ub tip begins to bifurcate, stromal cells continue to be excluded from direct contact with the tips, but come into contact with the ureteric bud at the position where bifurcation occurs (the cleft). The numbers of stromal cells increase in the cleft region as bifurcation continues until a new branch has formed where the stromal cells now occupy the space in between tips in their characteristic pattern (Fig. 1 A d, e, arrowheads). This suggests that stromal cells either play an active role in bifurcation by inducing cleft formation, or are passively excluded from contact with the tip portion of the ureteric bud. In this case, defects that disrupt branching would be expected to also result in alterations in stromal cell patterning.

**Figure 2:**
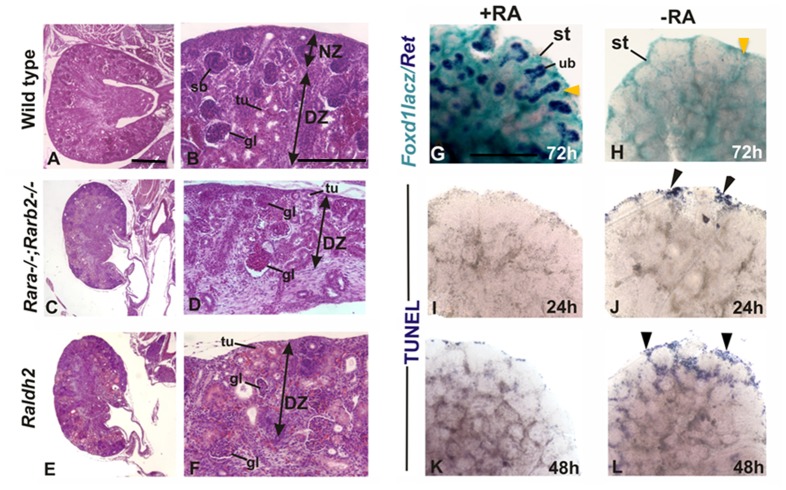
Retinoic acid controls stromal cell fate: A–F: Histological sections of wild type, *Rara*; *Rarb2* double mutant and *Raldh2* embryonic kidneys. A and B: sections of wild type kidneys showing normal distribution of the nephrons. NZ- nephrogenic zone: region under the renal capsule where continuous branching of ub and induction of nephron occurs. DZ- differentiating zone: region containing medullary stroma, differentiating nephrons and collecting duct branches. C, D and E, F: sections of *Rara^−/−^; Rarβ^−/−^* and *Raldh2* kidneys respectively showing reduced NZ region. In situ hybridization in cultured kidneys (G and H): *Foxd1LacZ* in vitro cultured kidneys probed for *Ret* expression. Expression of *FoxD1-lacZ* (blue) in the stromal cells of kidneys cultured in the presence of RA (G) or in the absence of RA (H) for 72 hours. Expression of *Ret* in the ureteric buds (shown in purple in G) is absent in kidneys cultured in the absence of RA (H). The expression of the stromal marker *Foxd1-LacZ* is shown in blue (G and H) in kidneys grown on RA+ or RA− media. I–L: wild type in vitro cultured kidneys; cultured in RA+ or RA− media. I and J: kidneys cultured for 24 hours and K and L kidneys cultured for 48 hours. Immunostaining with TUNEL to detect apoptotic cells (labeled in blue, I–L). Arrowheads in G and H indicate stromal cells, which are depleted in H. Arrowheads in J and L represent TUNEL positive cells which are increased in L.

To further address this, we used an organ culture system to examine the temporal sequence of branching defects and stromal cell abnormalities in kidney rudiments cultured without added RA, which in mutants lacking RA-receptor signaling, results in down-regulation of *Ret*, branching abnormalities and accumulation of stroma at the periphery of the kidney [15](Fig. 2 A–E). In the first set of experiments, kidneys from Hoxb7-Gfp embryos were cultured with or without RA and analyzed after 24 h, 48 h and 72 h to assess *Ret* expression and the branching (Fig. 1 B a–f and j–o). Stromal cell patterning was analyzed in parallel experiments in cultures of kidney rudiments from Foxd1-Gfp embryos grown with or without RA (Fig. 1 B g–I and p–r). Whole mount in situ analysis of Hoxb7-gfp organ cultures indicate that *Ret* expression which was abundant in controls at all times examined, was present in RA-depleted kidneys at 24 h, but declined to undetectable levels by 48 h (Fig. 1 B a–c and j–l) a finding that is consistent with the turnover rate of RA in tissues, which is 24–48 h. Analysis of the branching patterns in RA-containing and RA-depleted cultures revealed similar findings: unlike RA-containing cultures, rudiments cultured without RA displayed branching defects by 48 h; fewer ampullae could be distinguished and by 72 h, branching was arrested. In this case, individual asymmetric branches continued to extend and were angled laterally nearly touching one another, while in controls branches always extend outward at a 45 degree angle and never come into contact (Fig. 1 B d–f and m–o) (n = 5 per experiment).

We next compared the ontogeny of stromal cell abnormalities in rudiments from Foxd1-Gfp embryos cultured with or without RA. Analysis of rudiments after 24 h in culture revealed little difference in the distribution of Gfp+ stromal cells in RA-containing and RA-depleted cultures, however by 48 h, stromal cells which were present in the characteristic honeycombed pattern in controls, were diffuse at the periphery in RA-depleted kidneys, and did not occupy the normal position between branches. By 72 h, the stromal cell compartment was greatly depleted (Fig. 1 B g–i and p–r). These observations suggest that the distribution of cortical stroma is tightly correlated with the position of ureteric bud tips and branches. Depletion of the stromal compartment in cultures without RA suggests that retinoids may normally have a role in stromal cell survival (n = 5 per experiment).

### Retinoic Acid Controls Stromal Cell Fate

Analysis of E18 *Rarab2* and *Raldh2* mutants revealed down-regulation of *Ret*, defective branching and loss of the nephrogenic zone including the stromal compartment (Fig. 2 A–F). Analysis at earlier stages revealed that stroma was present, but had accumulated at the periphery of the mutant kidney, suggesting that RA signaling might normally be important for stromal cell survival [14]. To address this, we analyzed the distribution of stromal cells in kidneys from E14 Foxd1-LacZ embryos grown with or without added RA. After 72 h of culture, *Ret*, which was abundant in the control rudiments was undetectable in contralateral rudiments cultured without RA (Fig. 2 G, H), and these rudiments lacked the characteristic honeycombed pattern created by cortical stroma surrounding nephron progenitors and ub tips (Fig. 2H). To evaluate whether the abnormal pattern of stromal cells was due to apoptosis, we performed our analysis at earlier stages. Analysis after 24 h in culture revealed few apoptotic cells detectable by TUNEL staining in control kidneys cultured with RA (Fig. 2I), however in contralateral rudiments cultured without RA, apoptotic stromal cells were already detectable at the periphery of the kidney (Fig. 2J). The numbers of apoptotic cells increased by 48 h in RA-depleted kidneys, while only a small number of TUNEL-positive cells were detected in rudiments cultured with added RA (Fig. 2K, L). These observations suggest that RA may be important for stromal cell survival.

### Identification of a Stromal Cell Gene Expression Profile

#### Isolation of GFP-labeled stromal cells by FACS

To better understand the role of the stroma in kidney development, and to identify RA-regulated genes expressed in stromal cells, we used FACS to isolate Gfp+ stroma from E14 Foxd1-GFP kidneys cultured either with or without RA for 48 h or 96 h, then we performed expression profiling on RNA prepared from these samples using the GeneChip® Mouse Expression Set 430 for microarray analysis (Fig. S1). In all experiments we compared RNA from contralateral kidneys cultured with or without RA. The genes represented in the 6 arrays were normalized using Affymetrix Microarray Suite 5.0 (MAS 5.0). Normalized data were loaded into Genespring for visualization and further analysis. Differential expression was defined by 1 way ANOVA at a stringency of P<0.05. This analysis revealed 186 genes that changed more than 1.5 fold which were functionally annotated using DAVID V 2.1 beta (http://david.abcc.ncifcrf.gov/). Importantly, transcripts previously confirmed to be restricted to the cortical stroma such as *Foxd1, Alcam,* and *Rarβ,* were highly expressed, whilst few if any genes expressed in other renal compartments were detected, confirming that the stromal population was free of contamination with cells from other compartments. In addition, we identified a number of known RA targets including Retinal short-chain dehydrogenase/reductase 1 (*Sdr*), *Stra6* [21], [22] *Rarb*, [23] and *Rbp1* [24] (Table-1).

**Table 1:** Microarray data depicting stromal genes regulated by retinoic acid (>1.5 fold change in expression); also shown retinoic acid suppressed genes.

GENE	MGI Name	NCBI Ref Seq	Fold Change
Extracellular Matrix Protein 1	Ecm1	Nm_007899	9.6
Flavin Containing Monooxygenase 1	Fmo1	Nm_007672	7.04
Retinal Short Chain Dehydrogenase/Reductase	Sdr16c5	Nm_027301.3	7.86
Melanocortin 2 Receptor	Mc2r	Nm_008560	4.2
Wingless-related MMTV Integration Site 5A	Wnt5a	Nm_009524	4.17
Leptin Receptor	Lepr	Nm_010704.2	3.3
Stimulated By Retinoic Acid 6	Stra6	Nm_009291	3.28
Neurotensin Receptor	Ntsr 1	Nm_018766.2	3.17
GATA Binding Protein 6	Gata6	Nm_010258	2.92
CD83 Antigen	Cd86	Nm_009856	2.59
Retinoic Acid Receptor Beta	Rarb	Nm_011243	2.57
Cellular Retinoic Acid Binding Protein	Crabp1	Nm_013496	2.24
T-box 19	Tbx19	Nm_032005.4	2.12
Homeobox C4	Hox C4	Nm_013553	2.04
Retinal Short-chain Dehydrogenase/Reductase 1	Sdr16c5	Nm_027301.3	7.88
Cytochrome P450, Family 7, Subfamily B, Polypeptide 1	Cyp7b1	Nm_007825	6.05
Retinol Binding Protein 1, Cellular	Crabp1	Nm_013496	4.86
RA-SUPPRESSED			
Cellular Retinoic Acid Binding Protein I	Crabp1	Nm_013496	−9.53
Cytochrome P450, Family 1, Subfamily B, Polypeptide 1	Cyp7b1	Nm_007825	−4.65
Aldolase 3, C Isoform	Aldoc	Nm_009657.3	−3.13

We also compared the stromal markers to genes shown to be temporally dynamic or spatially restricted during kidney development. In each case we compared the stromal gene list to the lists of genes that were previously deemed significant and tested the chance of seeing the observed similarity by randomly selecting genes from the mouse genome. There was a strong concordance between the temporal lists described previously [25] plus enrichment in the non-ureteric populations (determined by profiling sorted HoxB7-GFP ureteric epithelium versus rest) and early metanephric mesenchyme (MM) and intermediate mesoderm (IM). Finally there was strong enrichment with kidney and bone marrow population gene lists.

### Verification of Microarray Data

**Figure 3:**
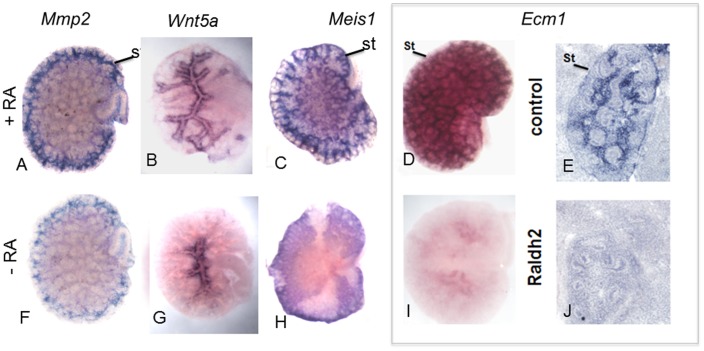
Validation of Microarray data. Kidneys cultured in the presence of RA (A, B, C) or absence of RA (F, G, H) and subjected to in situ hybridization of the various genes. Ecm1 is dependent on retinoic acid and regulates *Ret* expression in the ureteric bud clefts. *Ecm1* expression pattern in wild type (D wholemount and E section) and *Raldh2* kidneys (I wholemount and J section).

We validated our findings from the microarray experiment using in situ hybridization analysis of kidney rudiments grown with or without RA. These experiments identify a number of candidates including *Ecm1, Sdr1, Wnt5a, Mmp2* and *Meis1* which were selectively expressed in stroma whose expression was down-regulated in kidney rudiments cultured without added RA and were down-regulated in mutants lacking *Raldh2*, the major RA-synthesizing enzyme (Fig. 3 A–J and *Sdr1* not shown). *Ecm1* was the most highly induced gene observed in the array and previous studies reveal that, loss-of-function mutations in the human *ECM1* gene leads to two skin disorders: lipoid proteinosis, a rare autosomal recessive disorder and lichen sclerosis a common acquired disorder [26]–[28], and inflammatory bowel disease, both of which have been treated with retinoids [29], [30]. *Ecm1* is expressed in several tissues including skin, liver, small intestine, lung, ovary, prostate, testis, skeletal muscle, pancreas and kidney. Ecm1 is known to interact with several members of the extracellular matrix family [31] and a major heparan sulfate proteoglycan, Perlecan [32], [33]. Members of the heparan sulfate proteoglycan family are known to enhance the formation of receptor-ligand signaling complexes, they also bind cell surface ligands and regulate their turnover [34]. Based on the skin phenotypes of *ECM1* mutations it is hypothesized that *ECM1* serves as a scaffold bringing together structural proteins and growth factors in close proximity [35]. A role for retinoids as regulators of *Ecm1* has been demonstrated in recent studies. For example, in patients with Lipoid proteinosis, the extensive scarring of the skin due to accumulation of hyaline can be alleviated by retinoid treatment. The loss of function mutations in *Ecm1* in Lipoid proteinosis is thought affect its function in epidermal differentiation and basement membrane formation [36], [37]. In ulcerative colitis, an inflammatory bowel disease caused by mutations in Ecm1 is also treated with retinoids [30]. These observations led us to examine whether *Ecm1* may be a target of RA-signaling in the developing kidney, and may play a role in processes such as branching morphogenesis that may depend on Ecm1 remodeling.

### Ecm1 inhibition Results in Deregulation of *Ret* in the Ureteric bud and Impaired Branching

**Figure 4:**
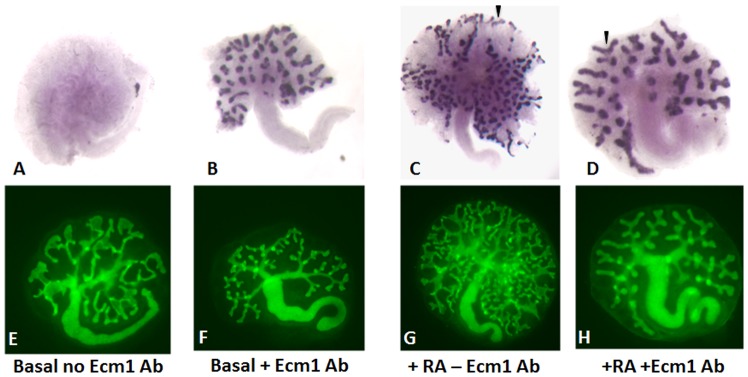
Blocking Ecm1 function alters *Ret* expression and branching. In vitro cultured kidneys from Hoxb7-GFP mice grown either in the absence (A, C, E and G) or in the presence(B, D, F and H) of the Ecm1 blocking antibody. A–D: Kidney rudiments were subjected to insitu hybridization and probed for *Ret* expression. E–H: Hoxb7-GFP kidneys showing branching of the ureteric bud tip. C and D: Arrowheads indicate the expression of *Ret* in the ub tips (C) or in the clefts (D).

To determine whether Ecm1 is normally important for kidney development, we tested whether inhibition of Ecm1 activity disrupts branching morphogenesis or other aspects of renal development. We used a blocking antibody (RB 469) that selectively labels Ecm1-expressing cells (Suppl. Fig. 2) and inhibits the ability of Ecm1 to interact with its partners [38]. The anti-ECM1 antibody (RB 469) was previously used to reveal the interaction/binding of COMP to ECM1 in human primary chondrocytes [38]. They demonstrated that the blockage of ECM1 function with antibody Rb469 (and also via siRNA) abolished the PTHrP mediated inhibition of Col X expression in vitro (Personal communication, Dr. Kong L). To determine whether Ecm1 was normally important for branching morphogenesis, Hoxb7-Gfp E12 kidney rudiments were cultured either in the presence or absence of RA and Ecm1 specific blocking antibody for 72 h, then analyzed to assess the branching pattern and *Ret* expression (n = 5 per experiment, from 5 independent cultures). Analysis of controls kidneys cultured with RA but without blocking antibody revealed that *Ret* expression was strong in the tips of ureteric bud and branching was robust (Fig. 4 C arrowheads) while in contralateral rudiments cultured with Ecm1 blocking antibody branch numbers were greatly reduced as was the size of the kidney (Fig. 4 B and D). Analysis of *Ret* expression revealed expression throughout ureteric bud tips, including the cleft domain where *Ret* is usually down-regulated (Fig. 4 B and D arrowheads, n = 4). These effects were abrogated by addition of oligopeptide PTRGTDANPAPGSKEE which blocks the inhibitory activity of the Ecm1 antibody, confirming that Ecm1-blocking activity of the antibody was specific (Fig. S2). Ecm1 is selectively expressed in cortical stroma, which is only in direct contact with the ureteric bud at the cleft, where *Ret* expression is normally down-regulated. That Ecm1 antibody blocks branching and expands the domain of *Ret* expression suggests that Ecm1 expression in stroma may be important for bifurcation of the ureteric bud by down-regulating *Ret* in the cleft domain. We observed that addition of the Ecm1 blocking antibody to kidneys cultured in basal media results in “improved” branching of the ureteric bud compared to control kidney rudiments grown in basal media without the Ecm1 blocking antibody (Fig. 4 A and B). Our observations suggest the presence of Ecm1 (or Ecm1-like molecule) in the culture that, when blocked by the anti-Ecm1 antibody leads to ectopic *Ret* expression in the ub clefts.

## Discussion

### Role of Retinoic Acid on Stromal Cell Fate

Analysis of kidney development in mutants with impaired RA-signaling has underlined the importance of this signaling pathway for normal development of the kidney [15], [19]. In the present study we examined the consequence of abrogated retinoid signaling on the distribution, maintenance and fate of the stromal cells and ureteric bud branching. In *Rarab2* double mutants and *Raldh2* mutants at E14, cortical stroma is present as an abnormally thick layer around the periphery of the kidney [14]. To investigate the cause of stromal abnormalities further, we followed the distribution of the stroma in organ cultures of *Foxd1-lacZ* kidneys cultured with and without RA. These studies revealed accumulation of stroma at the periphery of the kidney, however there was little evidence for migration of directed movement of stromal cells, suggesting that this abnormal accumulation is a consequence of abnormal branching morphogenesis: As the ureteric bud elongates and bifurcates nephrogenic mesenchyme remains associated with bud tips and the space in between the clefts of the bud are filled by the stromal cells.

Analysis of *Raldh2* and *Rarb2* mutants at birth revealed almost total absence of a nephrogenic zone, including cortical stroma, suggesting that stromal cells may have undergone apoptosis in the absence of RA-signaling. Our *in vitro* culture experiments with *Foxd1-lacZ* kidney rudiments grown in the absence of RA, we observed abundant apoptosis in the stromal compartment after 24 h in culture, which increased with time, suggesting that RA signaling may normally be important for regulating survival of the stroma. Similar roles for RA-signaling have been identified in a number of cell types where RA regulates survival by modulating expression of anti-apoptotic proteins [39] suggesting that RA signaling plays an important role in the survival of the stromal cells.

### Stromal Cell Signaling and kidney Development

Cortical stroma has been implicated in a number of events during renal development. For example stromal cells in the renal capsule are likely to be important for signaling that establishes the nephrogenic zone such as synthesis of retinoic acid, and may also prevent contact between cell types in the developing kidney with alien signaling molecules such as Bmp4 which inhibits branching morphogenesis [3] [9]. Our studies here identify a novel function for cortical stroma, as a source of extracellular matrix molecules that may normally be important for patterning of the ureteric bud. Whether Ecm1 acts alone in this process or in combination with other members of the Ecm family is an interesting question.

### Blocking Ecm1 Activity Results in Ectopic Ret Expression and Defective Branching

Our previous studies indicate that RA-receptor signaling in the ureteric bud is necessary for branching morphogenesis [19], [40]. Our previous studies suggest that RA acts in large part by maintaining expression of *Ret*, a gene required in the ureteric bud for branching morphogenesis [11], [15]. Our present studies suggest that RA-has an independent cell-autonomous role in cortical stroma, regulating expression of Ecm1. We show that cortical stroma are only in direct contact with ureteric bud at the cleft domain and are excluded from contact with tip cells, and we show that blocking Ecm1, which is selectively expressed in cortical stroma causes branching defects. That blocking Ecm1 abrogates the normal suppression of *Ret* expression in the ureteric bud cleft domain, suggests that RA-regulation of Ecm1 is normally important for cleft formation and ureteric bud bifurcation. Our studies show that the normal function of Ecm1 is to down-regulate *Ret* expression in the ureteric bud clefts, which may be important for normal bifurcation of ub and branching.

That we did not observe these phenotypes in previous studies is not surprising, since *Ret* expression was never present in kidneys of mutants, and branching defects were so severe that additional Ecm1-related defects would most likely be masked by the Ret-related defects. The possibility that cleft formation and bifurcation in the branching kidney are regulated by extracellular matrix deposition is consistent with observations from studies on other organs, where extracellular matrix is an important regulator of branching [20]. Further studies using an inducible model to inactivate RA-signaling in stroma, will be important for addressing these questions.

## Materials and Methods

### Mouse Strains

All mice were maintained in the Swiss-Webster genetic background. Embryonic day 0.5 was considered to be at noon on the day of the plug. Littermates were used for all experiments in which normal and mutant embryos were compared. The *Foxd1^LacZ^* mice were generated as previously described [3]. The *Hoxb7^GFP^* mice were a gift from Frank Costantini.

### Generation of *Foxd1-GFP* Transgenic Mice

*Foxd1-lacZ* mice were a gift from Eseng Lai [5]. *Foxd1^GFP^* mice were developed by replacing the *LacZ* coding sequence of the homologous recombination targeting vector used to generate the original *Foxd1^LacZ^* strain [5] with the *EGFP* coding sequence from pEGFP-N1 (Clontech). Linearized vector was electroporated into mouse ES cells and successful recombinants were screened for by antibiotic selection. Subsequent Southern blotting confirmed proper recombination at the *Foxd1* locus, after which these recombinant ES cells were injected into blastocysts from C57BL6/J females. One clone transmitted the *Foxd1^GFP^* deletion through the germ line and the resulting male progeny was used to outcross to wild-type Swiss Webster females. The experimental design used in these studies was approved by the institutional animal care and use committee at Columbia University.

### Isolation of Foxd1^ GFP^ Stromal Cells

Embryonic kidneys at E14 were dissected in ice-cold DMEM/F12 medium and culture on Transwell Clear filters (Costar) in serum-free medium with the following additives: insulin (5 µg/mL), transferrin (5 µg/mL), selenium (5 ng/mL), with pen/strep/glu antibiotic cocktail. The kidneys were incubated at 37°C in a 5% CO_2_ atmosphere for 2 days and 4 days. All-trans-RA (Sigma) and 9-cis-RA (Biomole) were added to the culture medium to give a final concentration of 1 µM. For the microarray analysis, one sample of kidney was cultured in serum-free medium with retinoids, and the control sample was cultured in the same medium but without the retinoids.

### Fluorescin-activated Cell Sorting (FACS)

Cultured kidneys were digested with 2 mg/mL Collagenase (Worthington S2P5950) in DMEM/F12 with 10% depleted FCS for 30 minutes at 37°, followed by incubation with 0.05% trpysin/0.5 mM EDTA for 5 minutes at room temperature. The kidneys were dissociated into cells by pipetting up and down for several times. After spinning for 5 at 800 rpm, cells were resuspended with PBS containing 0.5% depleted FCS and 1 mM EDTA at a final concentration of 2×10^6^ cells/mL and then filtered with 5 mL polystyrene round bottom tube with cell-strainer cap (Falcon). Propidium Iodide (Sigma) was added at a final concentration of 0.5 µg/mL to label the dead cells. FACS analysis was done with the FACS Vantage (Beckon Dickinson Immunocytometry Systems). Dead cells were excluded from the plots based on propidium iodide. The isolated cells were spun down at 1000 rpm for 5 minutes and resuspended in Trizol Reagent (Invitrogen).

### Total RNA Isolation and Purification

Using Trizol Reagent, total RNA was extracted from the sorted cells to create 5 pools of RNA from RA-treated stroma and non-treated stroma respectively. The total RNA was then purified with RNeasy Mini Kit (QIAGEN). All the RNA samples were stored at −80°C.

### cRNA Probe Generation and Hybridization to Affymetrix Microarray Chips

Total RNA samples were used to generate cRNA probes by two rounds of transcription (small sample protocol from Afymetrix). Basically, a poly (dT) primer (with the 5′ end carrying T7 promoter sequence) was used to synthesize cDNA from 100 ng total RNA. The cDNA were used to amplify cRNA using T7 polymerase. The cRNA product from this first round amplification was used to generate more cDNA by random priming, with the 3′ end carrying a T7 polymerase sequence. This cDNA was used to transcribe biotinylated cRNA which was used to hybridize to the mouse expression array 430 produced by Affymetrix following manufacturer’s protocol.

### Microarray Data Analysis

The Affymetrix mouse expression Array 430A was used to compare gene expression profiles of the GFP-positive stromal RA-treated and non-treated cells according to manufacturer’s protocol. Briefly, biotinylated cRNAs were synthesized and hybridized to the GeneChip probe arrays, which were then washed in the washing solution, stained with streptavidin-phycoerythrin and scanned. Analysis was made using Affymetrix GeneChip software.

### In situ Hybridization and Immunohistochemistry

We carried out non-radioactive in situ hybridization of sections as described [19] and whole-mount in situ hybridization as described [14]. ECM1a and ECM1b plasmids (gift from Dr. J.Merregaert) were linearized with SalI and Sp6 polymerase was used to generate 1.9 and 1.4 kb antisence Digoxin-labeled riborpobes. Wnt5a probe (gift from Dr. Jan), *Sdr1* cDNA (gift from Dr. Haeseleer F) was linearized with BamH1 and T7 was used to generate 2 kb probe.

In situ hybridization on frozen sections and whole mount tissue were performed using DIG- labeled probes as described previously [19]. *Six2* probe was a gift from the Costantini lab. The *Pax2, Wt1* and *Ret* probes have been previously described [19]. Anti- Ecm1 antibody (1∶50) was a gift from Dr. Merregaert. Anti-Ecm1 antibody was raised against the oligopeptide PTRGTDANPAPGSKEE, encoding the carboxy terminus of the ECM1a protein and purified as previously described [38], [41].

### In vitro Culture Experiments

Kidneys were isolated from embryonic day 11.5 or 12.5 embryos and placed on 0.45 μ transwell filters and grown in either basal medium, media containing RA or RA plus Ecm1 antibody. The kidney rudiments were cultured for 3 days and the fixed with 4% PFA. They were then passed through a sucrose gradient prior to cryo-sectioning or passed through methanol gradient prior to in situ hybridization experiments.

## Supporting Information

Figure S1 **The diagram of the** ***Foxd1-GFP*** **construct (A).** Fluorescent image of the Foxd1-GFP kidney expressing GFP in the stromal cells (B). FACS plot of the GFP tagged stromal cells (C).
(TIF)

Figure S2 **In vitro cultures of E12 kidneys grown in the presence of RA and anti-Ecm1 antibody or RA and anti-Ecm1 antibody plus blocking peptide.** E12 kidneys grown in basal media (A), E12 kidneys grown in the presence of RA plus anti-Ecm1 antibody (B), and (C) RA and anti-Ecm1 antibody plus blocking peptide. Expression of *Ret* in the clefts (arrows) in (B). Expression of *Ret* in the utreteric bud tips (arrows) in (C).
(TIF)
