# Supplementary figures and images for: Stromal Protein Ecm1 Regulates Ureteric Bud Patterning and Branching
Source: PLoS One. 2013 Dec 31;8(12):e84155. doi: 10.1371/journal.pone.0084155 (PMC3877229; doi:10.1371/journal.pone.0084155)

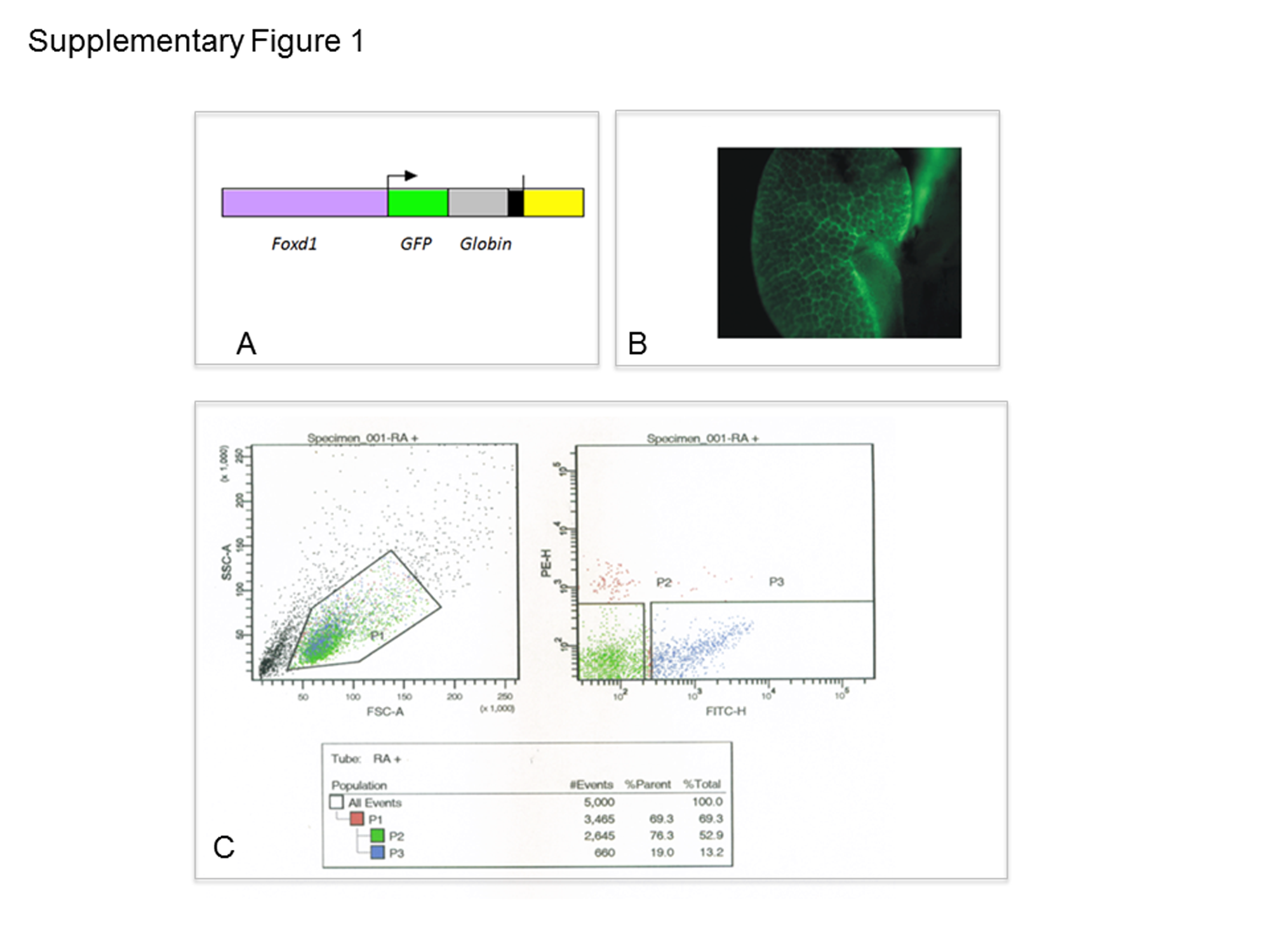

Supplement: Figure S1 — The diagram of the Foxd1-GFP construct (A). Fluorescent image of the Foxd1-GFP kidney expressing GFP in the stromal cells (B). FACS plot of the GFP tagged stromal cells (C). (TIF) [file pone.0084155.s001.tif]

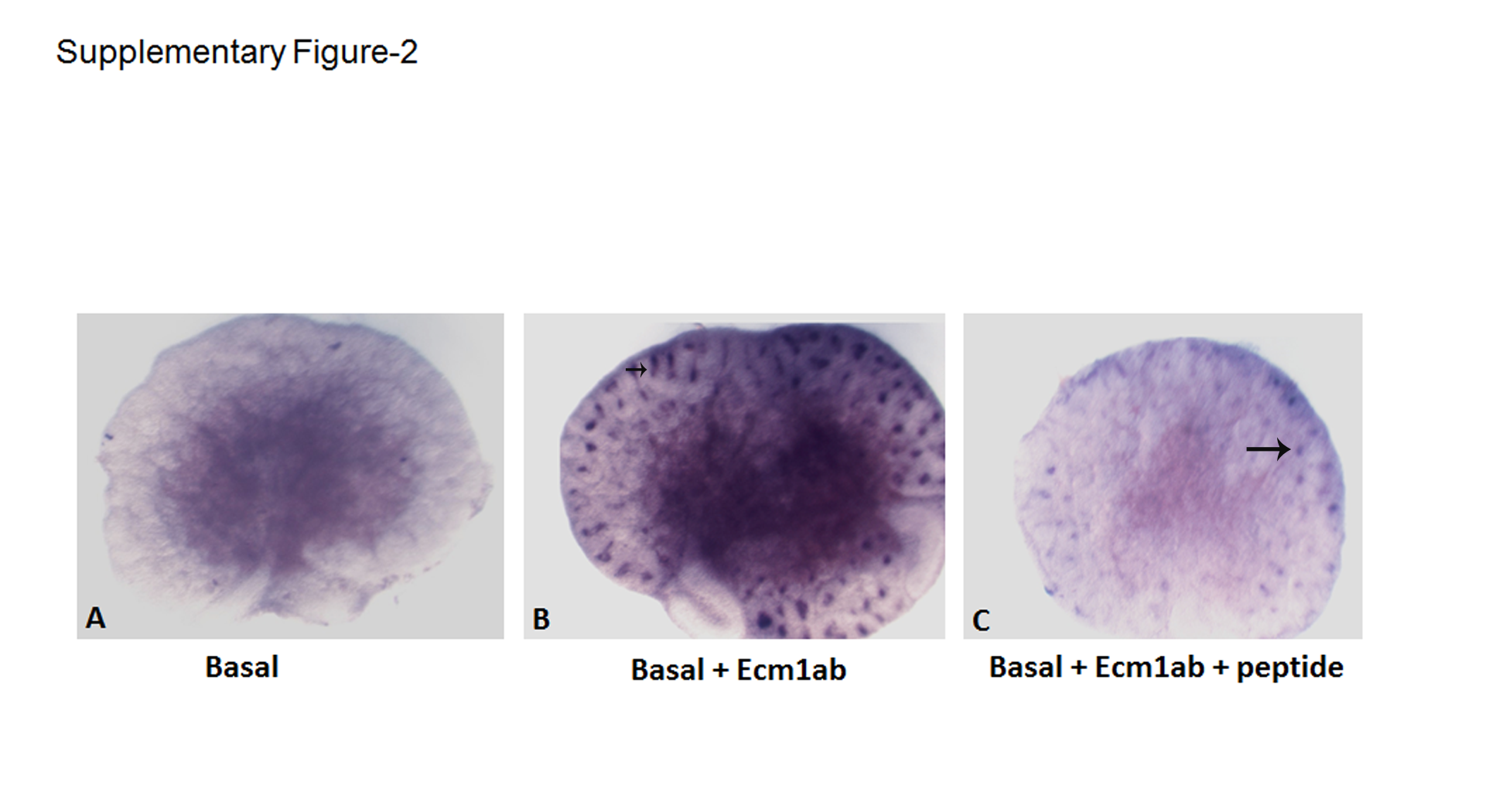

Supplement: Figure S2 — In vitro cultures of E12 kidneys grown in the presence of RA and anti-Ecm1 antibody or RA and anti-Ecm1 antibody plus blocking peptide. E12 kidneys grown in basal media (A), E12 kidneys grown in the presence of RA plus anti-Ecm1 antibody (B), and (C) RA and anti-Ecm1 antibody plus blocking peptide. Expression of Ret in the clefts (arrows) in (B). Expression of Ret in the utreteric bud tips (arrows) in (C). (TIF) [file pone.0084155.s002.tif]
